# Supplementary material for: Transcriptome Analysis Reveals Vimentin-Induced Disruption of Cell–Cell Associations Augments Breast Cancer Cell Migration
Source: Cells. 2022 Dec 13;11(24):4035. doi: 10.3390/cells11244035 (PMC9776984; doi:10.3390/cells11244035)
Supplement: Supplementary file 1 [file cells-11-04035-s001.zip › DEGs and enrichment analysis.zip!/DEGs and enrichment analysis/WT_VIMvsVector KEGG/ALL/WT_VIMvsVector_ALL.pathway_map.zip!/WT_VIMvsVector_ALL.pathway_map/src/hsa04976.html]

hsa04976


- hsa:8671

- Down regulated genes

ENSG00000080493(-3.03126070134589)

- hsa:107

- Up regulated genes

ENSG00000164742(1.87205666251591)

- hsa:10257

- Down regulated genes

ENSG00000125257(-3.20715697961916)

- hsa:10257

- Down regulated genes

ENSG00000125257(-3.20715697961916)

- hsa:6513

- Up regulated genes

ENSG00000117394(1.94254957435037)

Close
